# Role of Epigenetics in Biology and Human Diseases

**DOI:** 10.22045/ibj.2016.01

**Published:** 2016-11

**Authors:** Azam Moosavi, Ali Motevalizadeh Ardekani

**Affiliations:** 1 Department of Biochemistry, School of Medicine, Alborz University of Medical Sciences, Alborz, Iran; 2 National Institute of Genetic Engineering and Biotechnology, Tehran, Iran

**Keywords:** DNA modification, DNA methylation, Gene expression

## Abstract

For a long time, scientists have tried to describe disorders just by genetic or environmental factors. However, the role of epigenetics in human diseases has been considered from a half of century ago. In the last decade, this subject has attracted many interests, especially in complicated disorders such as behavior plasticity, memory, cancer, autoimmune disease, and addiction as well as neurodegenerative and psychological disorders. This review first explains the history and classification of epigenetic modifications, and then the role of epigenetic in biology and connection between the epigenetics and environment are explained. Furthermore, the role of epigenetics in human diseases is considered by focusing on some diseases with some complicated features, and at the end, we have given the future perspective of this field. The present review article provides concepts with some examples to reveal a broad view of different aspects of epigenetics in biology and human diseases.

## INTRODUCTION

Classical definition by Conrad Waddington in the 1950s states “an epigenetic trait is a stably heritable phenotype resulting from changes in a chromosome without alterations in the DNA sequence”[1]. Based on our understanding of epigenetics, actual epigenetic definitions express that the whole DNA content is exactly the same in somatic cells of one species, while gene expressions patterns have distinct differences in various cell types that can be clonally inherited[2]. Epigenetic mechanisms can influence the gene activity at the transcriptional and post-transcriptional levels and/or at the translation level and post-translational modifications. Such epigenetic mechanisms with a potentially vast spectrum of consequences could result in more varieties of cell differentiations, morphogenesis, variability, and adaptability of an organism, which can be affected by both genetic and environmental factors[3]. Therefore, the field of epigenetics covers the modifications of DNA, DNA-binding proteins, and histones, which are important in making changes in chromatin structure without any change in the nucleotide sequence of a given DNA. Also, some of these alterations could be transferred between generations[4].

### Epigenetic field and history

Following Fleming’s discovery of chromosome in 1879, Thomas Hunt Morgan demonstrated that there is a genetic linkage between several Drosophila genes and X chromosome. Other studies have assigned individual genes to specific sites on the Drosophila chromosomes. In 1930, H. J. Muller carried out further genetic analyses and introduced a class of Drosophila mutations, which were connected to chromosomal rearrangements. He concluded that “chromosome regions affecting various characters at once, are somehow concerned, rather than individual genes or suppositious ‘gene elements.”[2,5,6].

In the past few decades, many investigations have shown that the epigenetic mechanisms are involved in regulation of all biological process in the body from conception to death. These functional mechanisms are involved in genome reorganization, early embryogenesis and gametogenesis, as well as cell differentiation. The interplay of DNA methylation and histone post-translational alterations, which cause as the result of regulatory proteins and non-coding RNAs, are key epigenetic players to rearrange chromatin into areas such as euchromatin, heterochromatin, and nuclear compartmentalization. Epigenetic signs may have long-term impressions, for instance, in learning and organizing memory or predispositions to different cancers. Incorrect epigenetic marks can result in birth defects, childhood diseases, or symptoms of diseases in other interims of life. Epigenetic mechanisms also regulate development and adaptations during the life of an organism, and their alterations may result in various disorders such as cancer. On the other hand, some epigenetic marks can be reversible, and this fact has encouraged many researchers to focus on epigenetic therapy[7]. In recent years, it has been demonstrated that DNA methylation, in some cases, can be irreversible[7-9]. This trait could be useful in complex features and challenging diseases such as memory function, psychological behaviors and injuries, addiction, cancer, and other diseases that could not be explained just by genetic factors or the environment.

### Epigenetic modifications

**Fig. 1:**
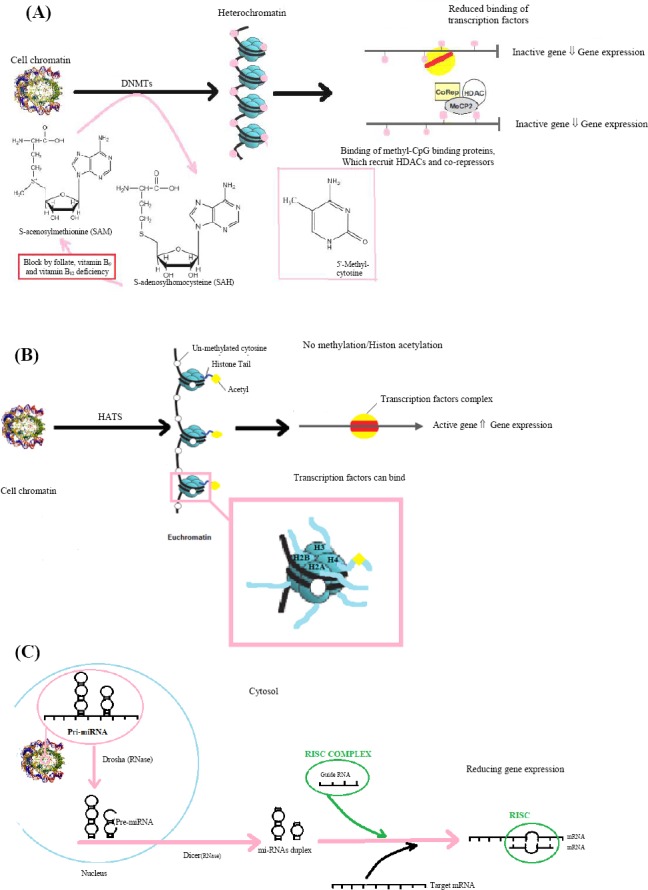
Three major epigenetic modification mechanisms. A) DNA methylation. DNA methylation is mediated by DNA methyltransferase enzymes at CpG sites. It can also decrease gene expression by reducing the binding of transcription factors or increasing the binding of methyl-CpG binding proteins[11,12,59,100]. B) Histone modification. Histone acetylation, particularly in lysine residues of histone tails, is an important histone modification that can accelerate binding transcription factors and then gene expression beside DNA demethylation[18,59]. C) miRNA. The formation of miRNA begins in nucleus and continues in cytosol that can perform a mechanism to regulate gene expression in mRNA level[101].

In a multicellular organism, the epigenetic changes enable different adult cells to express specific genes that are required for the existence of each cell type and transfer of information to the daughter cells. Epigenetic modifications often happen during an organism’s lifetime; however, these changes can be transferred to the next generation if they occur in germ cells[10]. Paramutation, bookmarking, imprinting, gene silencing, X chromosome inactivation, position effect, changeable disorder or phenotypic severity, reprogramming, maternal attributes, carcinogenic processes, teratogenic effects, regulation of histone modifications, heterochromatin states and cloning are known to involve epigenetic processes. Three major epigenetic modification mechanisms are shown in Figure 1.

### DNA methylation and demethylation

DNA methylation status has high stability and serves as a special epigenetic memory of specific cells throughout all periods in the cell cycle. It may also regulate the expression and the activity of histone codes. Acceleration of DNA methylation at CpG sites is mediated by DNA methyltransferase enzymes such as DNMT1, DNMT3a, and DNMT3b. Inside the cells, S-adenosyl methionine act, as an important methyl group donor. In this sense folic acid and B12 play the determinant roles in re-methylation or the attraction of de-methylated form of S-adenosyl methionine through passive and active mechanisms[11,12]. A number of studies have demonstrated that these effective factors could change DNA methylation patterns and alter the levels of gene expression[13,14]. Studies have also confirmed that the nutritional status in the early years of life could affect DNA methylation pattern and gene expression levels in adulthood[15]. Furthermore, the methylation patterns in CpG sequences at cytosine residues can be heritable and act as tissue- and species-specific features. It is interesting that 70% to 80% of human DNA in CpG sequences are usually methylated[9,16], and correlations between methylation and expression levels in *cis* and *trans* have been reported[17]. Totally, DNA methylation, as a very impressive epigenetic agent, could influence the development of mutations, DNA faultless and durability, gene expressions, and chromatin modifications.

### Histone and non-histone modifications

Histone modification is one way of gene regulation through chromatin remodeling and includes acetylation, methylation, phosphorylation, ribosylation, ubiquitylation, sumoylation, and citrullination. Acetylation has been studied frequently in different investigations, and it has been shown to be mediated by five families of mammalian histone acetyltransferase enzymes[18]. Another group of molecules, known to act as non-histone modifiers, is RNA transcripts, which are necessary to maintain the activity of genes (directly or indirectly). For example, hepatocyte nuclear factor 4 increases the special gene transcription level, and MyoD has similar effects on muscle-specific genes[19]. miRNAs are about 17 to 25 nucleotides and are considered as a member of the non-coding RNAs that can mediate a large number of biological activities[20]. It has been demonstraed that the expression of miRNAs in a cell can regulate functions of about 200 messenger RNAs as their targets[21]. miRNAs can also regulate about 60% of protein-coding genes in human[22], and many miRNAs are epigenetically adjusted by methylation in CpG islands or histone modifications or both of them[20,23].

Structural inheritance is another non-histone mechanism of inheritance. Experimentally, it has been shown that altered cellular patterns are inherited to the next generation of cells, and it appears that the present structures act as templates for new structures[24].

Also, evidence has confirmed the importance of positioning in addition to epigenetic modifications such as DNA methylation and hydroxymethylation, which play an important role in structural inheritance[25].

### Mitotic gene bookmarking

An important issue in interpreting epigenetic and genetic modifications is the concept of mitosis gene bookmarking. Mitosis can play an important role in remodeling the transcriptional landscape. This view confirms that bookmarking mechanisms provide flexibility to permit alteration in cellular fate or differentiation. The distinct kinetics of dissociation and re-association of factors during mitosis as well as varying stabilities of histone marks argue that multiple mechanisms control mitotic partitioning[26].

Mitotic chromatin is transcriptionally inactive[27] and is excluded from most of the factors such as transcription agents and RNA polymerases[26,28-32]. To correct and complete cell division, regulatory proteins should re-use their exact genomic targets to return gene transcription states appropriately[26,33-36]. Studies have shown that the exact post-mitotic restoration of suitable transcriptional patterns is affected by epigenetic marking of mitotic chromatin to prevent tragic regulatory results[37,38]. For example, certain histone and DNA modifications remain in mitotic chromatin [39-42]. DNA methylation keeps transcription silent until the completion of mitosis[23,43], whereas specific histone modifications do not show such a clear effect on gene expression, and there are more elusive states and relations. After mitosis, to reactivate different sites of genes, transcription factors have to find their appropriate sites, which are located in transcriptionally silent chromatin through mitosis process[26]. For instance, poly ADP-ribose polymerase-1 creates stable epigenetic marks in metaphase chromatin at the transcription start sites of many genes that are necessary to restart transcription after mitosis[28].

### Role of epigenetics in biological processes

Epigenetic modifications are a dynamic process reflecting a complex interplay between an organism and its environment. For example, the acetylation of lysine residues in histone subunits usually can result in promoting gene transcription, or the methylation of Lys9 or Lys27 of histone H3 is correlated with gene repression. However, the methylation of Lys4, Lys36, or Lys79 of H3 is ordinarily associated with gene activity[44]. The heritable quantity of gene expressions is about 30%, while this percentage is decreased to about 23% in cells grown in culture. Such associations between methylation levels and genetic variations have been demonstrated in several organisms, tissues, and populations[17,45,46].

Animal studies in rats[7] have shown that hippocampal-dependent contextual learning can elicit a remarkably persistent fear-related memory, and this behavior is dependent on *denovo* DNA methylation. The inhibitors of DNMTs could abolish fear-related memory by preventing DNA methylation. Other studies have suggested that histone acetylation and DNA methylation influence memory formation, and DNA methylation has a key role in the storage of long-term memories in cortical brain regions[7,17].

The role of methylation in biological clock has been an interesting discovery in recent years. Studies of 353 epigenetic markers (DNA methylation of CpG dinucleotide) in DNA have made it possible to predict the ageing of tissues[47]. Also, the role of methylation and acetylation during differentiation has been demonstrated by studying Oct4 promoter regions[48].

### Epigenetics and environment

Epigenome generally comprises all epigenetic modifications such as DNA methylation and histone modifications, as well as non-coding RNAs at any given point in time. The cell epigenome is dynamic and can be affected by genetic and environmental factors. Furthermore, epigenetic modifications can be reversible, which makes the genome flexible to respond to environment changes such as nutrition, stress, toxicity, exercise, and drugs[17].

In the winter of 1944/45 during World War II, food supply was reduced due to blocked food transport by Germans, and food delivery by boat was impossible because water canals were frozen. Famine and lack of sufficient vitamins and proteins in diets affected the whole population, especially pregnant women. Since then studies have been carried out on the long-term effects of this incidence on newborn babies and individuals under such conditions. The results of these studies showed that individuals born after the famine had a higher susceptibility to a subset of diseases, including schizophrenia, stress sensitivity, and obesity[17].

One of the nutritional components in food, which plays a major role in methylation, is folate. Folate can influence methionine production by homocysteine remethylation in the form of 5-methyltetrahydrofolate. It has been reported that folate defect or shortage can enhance colorectal carcinogenesis through hypomethylation of genomic DNA[12].

Stress is an important environmental factor. Recently, some studies have demonstrated that people with post-traumatic stress disorder, who were abused during childhood, exhibit different levels of DNA methylation and gene expressions in comparison to those who were not abused[49,50]. Also, maternal stress during gestation has been indicated to be associated with neurodevelopmental and psychiatric disorders. Long-term studies on children exposing to stress in utero have shown to be predisposed to psychiatric disorders because of an increase in the promoter activities of glucocorticoid receptor[17,51,52].

As a human, we are exposed to various environmental toxins on a daily basis, and this can affect our health through changes in our epigenome. *Listeria monocytogenes*, *Clostridium perfringens*, and *Streptococcus pneumonia* have been shown to induce dramatic changes in acetylations of histones via the toxins they produce[53,54].

Arsenic exposure studies have been demonstrated to result in global DNA alterations and gene promoters methylation levels, histone acetylation, histone phosphorylation, and miRNA expressions. Such influences of arsenic exposure have been linked to epigenetic dysregulation and carcinogenesis[55].

One of the major effects of physical exercise is on epigenetic modifications that can be beneficial to health and cancer patients. Modifications in DNA methylation patterns as a result of physical exercise can increase the expression of genes involved in tumor suppression and decrease the expression levels of oncogenes. Studies have shown that DNA methylation patterns are different in cancer cells, and hypermethylations and hypomethylations have been observed in the promoter of tumor-suppressing genes and oncogenes. These modifications could result in uncontrollable growth leading to tumorigenesis[56,57].

In patients with type II diabetes, several genes have been reported to be hypermethylated in muscle, including peroxisome proliferator-activated receptor gamma and coactivator 1-alpha[58,59]. Some drugs, such as procainamide and hydralazine have been shown[60] to have an enhancing effect on antinuclear antibodies. In recent studies, it has been reported that women using oral contraceptive pills have a lower global DNA methylation levels when compared to those who do not use such pills[61].

### Epigenetics and human diseases

Methylation is a common and widely used mechanism for epigenetic modifications in cells. It has been shown to be correlated with many human diseases, including different cancers, autoimmune disorders, neurological disorders (Fragile X syndrome as well as Huntington, Alzheimer, and Parkinson diseases and schizophrenia). Also, it has been suggested that methylation can be considered for complicated diseases influenced by some secondary factors such as sex differences and age, which could change disorder severity[62].

### Cancer

Epigenetic modifications have a considerable effect on cancer. Hypermethylation of promoter regions in tumor suppressor genes can inactivate many tumor suppressor functions. Methylation levels also play an important role in cell divisions, DNA repair, differentiation, apoptosis, angiogenesis, metastasis, growth factor response, detoxification, and drug resistance[12]. Such features have promoted huge advances in the early detection of cancer using methylation levels. For example, hypermethylation of promoter regions in *APC* and *RASSF1A* genes are considered as common epigenetic markers for early detection of cancer[63]. Also, hypermethylation of TP53 promoter region has been reported as a common marker for evaluation of cancer development[64]. There are also some other types of epigenetic changes in cancer. In recent years, dysregulation of miRNAs has been confirmed in breast cancer, which has a potential to be used as diagnostic biomarkers[65]. Also, hyper- and hypo-methylation of several genes in breast cancer have been confirmed[66].

Microsatellite instability, chromosomal instability, and CpG island methylator phenotype have been identified as three major mechanisms affecting gene function in colorectal cancer (CRC). Microsatellite instability occurs in 15% of CRCs, which can result in instability phenotype by mutated or methylated mismatch repair genes[67].

In a comprehensive analysis of CRC tumors in Iranian patients, Brim *et al*.[68] demonstrated a high microsatellite instability rate (18%). From 15 known methylation target genes, *APC2, PTPRD, EVL, GPNMB, MMP2*, and *SYNE1* were found to be methylated in most samples, which can be potentially used as specific clinical and pathological markers of CRC in this population[68].

The pathogenesis of CRC has been reported to be controlled by miRNAs, which can act as regulators of oncogenic and tumor suppressor pathways, responsible for the development of cancer. It has been confirmed that different miRNAs can be useful as biomarkers and are potentially applicable in prognosis evaluation and the detection of CRC stages[65]. It has been also observed that in the absence of O6-methylguanine-DNMTs activity as a DNA repair protein, the specific genes, such as *K-ras* and *p53*, might be accumulated by G-to-A transition. Furthermore, hypermethylation near the methylguanine-DNMT start codon in the specific locus is critical for cancer progression, which may have a prognostic value in CRC patients[69].

It has been indicated that miRNAs play an important role in many types of cancer: acute myeloid leukemia, acute lymphocytic leukemia, chronic myeloid leukemia, chronic lymphocytic leukemia, endometrial carcinoma, gastrointestinal cancer, lung cancer, bladder cancer, thyroid tumors, and esophageal adenocarcinomas. Hence, the potential applications of miRNAs in diagnosis and prognosis of these cancers would be highlighted in the near future[65].

Isocitrate dehydrogenase 1 (*IDH1*) and *IDH2* genes are frequently mutated in low-grade gliomas, *denovo* acute myeloid leukemias in adult and in the subsets of chondrosarcomas and lymphomas. Interestingly, high correlation between histone and DNA methylation phenotype in IDH mutant gliomas has been reported[18]. In Tables 1, 2, and 3, epigenetic modifications in different types of cancer are shown.

**Table 1:** Promoter methylation in different types of cancer

Cancer type	Gene	Promoter methylation	Reference
Breast	RARB2, MSH2, ESR1B, AKR1B1, COL6A2, GPX7, HIST1H3C, HOXB4, RASGRF2,TM6SF1, ARHGEF7, TMEFF2, RASSF1, BRCA1, STRATIFIN, RASSF1A	Hypermethylation	[102]
Gastric	RUNX3	Hypermethylation	[102]
Liver	CDKN2A	Hypermethylation	[102]
Esophageal	APC	Hypermethylation	
Colorectal	SEPT9, hMLH1, CDKN2A/p16, HTLF, ALX4, TMEFF2/HPP1, NGFR, SFRP2, NEUROG1, RUNX3,UBE2Q1	Hypermethylation	[103,104]
Lung	RARB2, RASSF1A, CHFR, STRATI-FIN, SHOX2, RASSF1A APC1	Hypermethylation	[102]

**Table 2:** Histone modifications in different types of cancer

Cancer type	Type of histone modification
Lung adenocarcinoma	Up-regulation of α-2 glycoprotein 1 in consequence of global histone acetylation[105]
Non-small cell lung	Global H3 deacetylation[106]
Gastric	Global H3K9 trimethylation[107]
	Silencing of RUNX3 in the consequence of increased H3K9 dimethylation and decreased H3 acetylation[108]
Prostate	Global H3K9, H3K18, and H4K12 acetylation and H4K3 and H3K4 dimethylation[109] Activation of PTEN, CYLD, p53, and FOX03a by modulating histone H3K9 methylation and deacetylation[110]
Colorectal	Global H3K9 deacetylation[111]
Pancreatic	Acetylation of histone H3 promoter region of C/EPBα[112]

**Table 3:** miRNA changes in different types of cancer[3,65,113]

Cancer type	Types of miRNA[ (+)=up-regulation/(-)=down-regulation]
Oesophageal squamous cell carcinomas	miR-21(+)
Lung	miR-17-92 (+)
	miR-34c, miR-145, and miR-142-5p, let-7(-)
Primary head and neck squamous cell carcinoma	miR-1, miR-133a, miR-205, and led-7d(-)
	bsa-miR-21(+)
Gastric	miR-106a(+)
	miR-433 and miR-9(-)
Prostate	miR-135b and miR-194(+)
	miR-23b, miR-100, miR-145, miR-221, miR-222(-)
Melanoma	miR-182(+)
Hepatocellular	miR-18a(-)
Colorectal	miR-let 7g, miR-21, miR-20a, miR-17-19 family, miR31, miR 135, miR-181b, and miR 200c (+)
	miR-34, miR-let7, miR-143, miR-145, miR-133b, and miR-126(-)
Bladder	miR-2 23, miR-26b, miR-221, miR-103-1, miR-185, miR-23 b, miR- 203, miR 17-5p, miR-23, miR-205(+)
	miR-29c, miR-26a, miR-30c, miR-30e-5p, miR-45, miR-30a-3p, miR-133a, miR-133b, miR-195, miR-125b, and miR-199a (-)
Breast	miR-21, miR-155, miR-23, and miR-191(+)
	miR-205, miR-145, miR-10b, and miR-125b (-)

### Autoimmune diseases

Natural and normal functions of immune system depend on self-tolerance, and self-tolerance deficiency can result in autoimmunity. Autoimmune disease concordance studies in both monozygotic and dizygotic twins have suggested a role for epigenetic factors. Epigenetic homeostasis failure, as a response to environmental agents, can result in gene expression changes in specific differentiated cells leading to dysregulated self-tolerance[70].

The immune system and target organ are two main players in an autoimmune disease process and the epigenetic modifications of these players could have roles in disease development. Many functions of immune cells such as hematopoietic lineage, rearrangement of antigen-receptor, allelic exclusion, and inducible immune responses against pathogens are epigenetically controlled. The alterations of epigenetic mechanisms regulating immunological development could promote autoimmunity disease[71].

Interestingly, the frequency of autoimmune disease occurrence is notably more in women, and the reason may be due to female sex hormones. The involvement

of second X chromosome in immune response and genetic predisposition to autoimmunity is suspected. Considering the lack of enough knowledge on the exact cause of these immune diseases, a role for epigenetic regulatory mechanisms is highly possible[70]. Furthermore, there are many examples for correlation between epigenetic modifications and autoimmune diseases. For example, in patients with rheumatoid arthritis, DNA hypomethylation of HDAC1 (histone deacetylase 1) and HDAC2 levels, hyperacetylation of histones H3 and H4, and hypomethylation of histone H3 at lysine 9 have been observed in synovial tissues. In addition, in patients with multiple sclerosis, the hypomethylation of DNA have been detected in central nervous system white matter in comparison to healthy individuals. In systemic lupus erythematosus, the main targets of autoantibodies are hypomethylated apoptotic DNA and modified histones[71].

Several studies have confirmed the role of epigenetics in allergic conditions, and asthma is considered as one of the most complicated diseases in this category. Evidence suggests that both asthma and epigenetic mechanisms are heritable, and 36–79% of heritable, familial asthma cases have non-Mendelian inheritance pattern in more than 100 genes[72-75], which covers only a small portion of the disease etiology[73]. Interestingly, asthma and epigenetic modifications have been shown to be transferred from affected mother more than affected father in parental origin features[76], which can be a result of immune interactions between the fetus and the mother[77]. Utero exposures can affect asthma as well as epigenetic modifications, and both features can be influenced by environmental factors[78,79].

Classically, allergens are considered in relation to factors such as smoking behavior[79-81], and studies have confirmed that these agents can change epigenetic marks in asthma[82].

### Neurodegenerative and psychological disorders

The parental allele-specific gene expressions along imprinted domains are brought about by specialized sequence elements called ‘imprinting control regions’ (ICRs). ICRs are located just on one of the parental copies whose function is regulating gene expressions through an allele-specific manner. Although DNA methylation is the best investigated epigenetic alteration at ICRs, methylation and acetylation of histones in ICRs have also been reported[83]. For putting methylation imprints onto the ICRs, DNMT3A is essential. After fertilization, through somatic maintenance, the allelic methylation changes are conserved during development. This process is complicated due to its link to the cell cycle and requires the proportional functions of multi-enzymatic complexes that could be affected by intrinsic and extrinsic agents[84]. Other important methylation modifications have been recognized in genes involved in the development of Alzheimer’s disease and schizophrenia. Distinct reduction in DNA methylation has been identified[85] in Alu and other repetitive elements in the genome that are exclusively related to the early phase of life. Also, a role for epigenetic modifications has been confirmed in psychiatric diseases such as Rubinstein-Taybi syndrome and addiction, Huntington’s disease, and Fragile X syndrome. It is now widely accepted that for normal function and neurodevelopmental features of the brain, the constancy of DNA methylation and histone modifications is essential, and their dysregulation may result in disease phenotypes[86].

The significant role of epigenetics in brain development and disease is due to the following factors: 1) plasticity of epigenetics during all periods of brain development and aging as well as dynamic regulation in neurons, 2) disordered chromatin organization in both early childhood and adult neurodegenerative disorders, and 3) rapid increase in chromatin modifying drugs demonstrated to have unexpected therapeutic potential for degenerative and functional disorders of nervous system. These factors have attracted a vast interest in chromatin-associated mechanisms of neurological diseases, and a new field of study called ‘neuroepigenetics’[87] have been established.

Numerous reports have pointed the association of DNA methylation with neurodegenerative diseases. Regulation of H3K4 methylation proteins are considered as an influencing factor in neuro-degenerative disease, and the inactivation of histone demethylase enzymes can result in different disorders such as autism, Rett syndrome, and X-linked mental retardation[88]. In Table 4, some mental and neurological disorders are listed alongside their epigenetic aberrations.

**Table 4:** Summary of epigenetic aberrations reported in mental diseases

Disease	Epigenetic change (tissues)	Ref.
Fragile X syndrome	Hyper-methylation at the FMR-1 gene with an expanded (CCG)n repeat	[11]
Huntington	Histone modification in HDACs and histone KDM5D/Kdm5d	[88]
Rett syndrome	Mutation in the gene encoding *MeCP2*	[11]
Autistic patients and their parents	Abnormal trans-methylation, trans-sulfuration metabolism, genome-wide DNA hypo-methylation and elevated blood homocysteine level (blood)	[11]
Down syndrome	miR-99a, let-7c, miR-125b-2, miR-155, and miR-802 up-regulation	[113]
SCZ	DNA hyper-methylation of the RELN promoter and SOX10 promoter (brain)	[11]
SCZ and BD	DNA hypo-methylation of the MB-COMT promoter (brain)	[11]
SCZ	Histone 3 lysine 4 hypo-methylation at the GAD1 promoter due to mixed- lineage leukemia 1 gene dysfunction (brain)	[11]
SCZ (male)	DNA hyper –methylation of the WDR18 gene (brain)	[11]
SCZ (male)	Global DNA hypo-methylation (blood)	[11]
SCZ & Psychotic BD	DNM T1 hyperexpression and increase in SAM content (corticalinter-neurons)	[11]
Bipolar ll	DNA hypo-methylation of *PPIEL* gene (blood)	[11]
BD (female)	Hypo-methylation of *RPL39* (brain)	[11]
Dementia	Hyper-methylation of circadian genes, *PER1* and *CRY1* (blood)	[11]
Alcoholism	DNA hyper-methylation of alpha synuclein promoter, HERP gene promoter and dopamine transporter gene (blood)	[11]

SCZ, schizophrenia; BD, bipolar disorders

### Addiction

The worldwide estimation of taking opioids (opium-like substances) is about 13.5 million people of which 9.2 million use heroin[89]. Family, twin, and adoption studies have presented a large amount of evidence that indicates genetic backgrounds have an important role in addiction disease. A Two- to four-fold difference in types of substance abuse disorders have been reported in monozygotic in comparison to dizygotic twins and also different genetic variations have been linked to the various types of substance dependency[90].

An interesting picture emerged from most of drug addiction genetic studies emphasizes the importance of environment factors besides genetic determinants. Epigenetic changes the responses to the needs of an organism in diverse environmental conditions through adaptive alterations. Epigenetic events and processes are usually used to incorporate the maintenance of neuroplastic changes, which are correlated with learning and memory[91]. Dysregulation of epigenetic machinery, as the results of substance exposures, can lead to drug-seeking behavior and relapse of substance dependence. For instance, neuronal activation can alter DNA methylation of the Brain-derived neurotrophic factor (BDNF) promoter at cAMP response elements (CRE) binding sites inducing gene expressions[92]. The crucial role of BDNF was bolded in neural and behavioral plasticity in chronic opiate exposure through a steady down-regulation of exon-specific *Bdnf* expression in the ventral tegmental area. Special epigenetic changes, such as histone modifications, have been reported to mediate *Bdnf* gene activities in chronic morphine exposure[93,94]. Other observations in rats during forced abstinence from morphine have shown a significant H3 acetylation increase in the BDNF promoter II and histone H3 methylation changes in ventral tegmental area[95]. Other studies have demonstrated that the prolonged drug exposure leads to widespread transcriptional changes of genes with diverse cellular functions. This type of change is different from the changes observed in the early stages of drug-induced neural adaptive processes accompanied with specific changes in early response genes and signal transduction pathways[96].

Altogether, studies have indicated that the etiology of initiation, continuation, and relapse of substance dependence will be better understood considering epigenetic factors, which regulate multiple interacting neural signaling pathways that create enormous diversity in the continually developing brain[97,98].

In this review, we presented and discussed the results of many studies demonstrating that epigenetic mechanisms regulate gene expressions in different models and at various levels. The importance of epigenetics in different human disorders have attracted many interests in the last decade, especially in complicated disorders such as behavior plasticity, memory, cancer, autoimmune disease, addiction as well as neurodegenerative and psychological disorders. It is becoming moreclear why many therapeutic approaches have failed in the past. It is hoped that by understanding epigenetic mechanisms involved in neurological and psychological disorders, more effective therapies would soon become available.

Because of great potential, academia and industry have shown great enthusiasm to develop new epigenetic therapies. Drug development based on epigenetics is difficult and expensive like other novel drug targets. However, reversible nature of epigenetic modifications has made therapeutic applications a possible alternative approach in the near future. DNA methylation inhibitors act against various cancers and also psychiatric diseases such as schizophrenia and bipolar disorders. For example, azacytidine and decitabine, as DNMT inhibitors, modulate epigenetic effects while toxicity and limited chemical stability of these drugs restrict their use in cancer therapy. In addition, histone deacetylase (HDAC) inhibitors are used in cancer therapy, cardiac hypertrophy, and heart failure and have been indicated to possess neuroprotective effects on cellular and animal models of Parkinson’s disease. In oncology, RNAi plays an important role as a target of epigenetic drugs. For instance, in human CRC, the upregulation of miRNA-135b is common, while in cervical cancer, miR-21, miR-126, and miR-143 are commonly upregulated[99].

Recent new data and knowledge relating to the importance of epigenetics in different human disorders promise a vibrant future for epigenetics research. The new research will integrate high-throughput sequencing technologies and the sophisticated algorithms to analyze the large amount of data produced by sequenced epigenomes. The epigenomic data will provide a chance to discover new epigenetic marks and their functions in different types of tissues, early development, and disease states. The association of epigenetic marks with specific diseases can help the development of tools to diagnose patients and measure the severity of a disease. Although there are issues with specificity and efficacy of many drugs being tested in animal models, further research on the epigenetic mechanisms will surely help the development of better therapeutic pathways and agents in the near future.
